# Linked Clinical Trials – The Development of New Clinical Learning Studies in Parkinson’s Disease Using Screening of Multiple Prospective New Treatments

**DOI:** 10.3233/JPD-139000

**Published:** 2013-01-01

**Authors:** Patrik Brundin, Roger A. Barker, P. Jeffrey Conn, Ted M. Dawson, Karl Kieburtz, Andrew J. Lees, Michael A. Schwarzschild, Caroline M. Tanner, Tom Isaacs, Joy Duffen, Helen Matthews, Richard K.H. Wyse

**Affiliations:** a Center for Neurodegenerative Science, Van Andel Institute, MI, USA; b Cambridge Centre for Brain Repair, Cambridge, UK; c Vanderbilt Center for Neuroscience Drug Discovery, Vanderbilt University Medical Center, Nashville, TN, USA; d Johns Hopkins University, Institute for Cell Engineering, Baltimore, MD, USA; e University of Rochester Medical Center, Center for Human Exp. Therapeutics, Rochester, NY, USA; f Reta Lila Weston Institute of Neurological Studies, University College London, London, UK; g Department of Neurology, Massachusetts General Hospital, Boston, MA, USA; h The Parkinson’s Institute and Clinical Center, Sunnyvale, CA, USA; i The Cure Parkinson’s Trust, UK. The Pavilion, Mickelfield Hall, Sarratt, Herts, UK

**Keywords:** Drug repositioning, disease modification, neuroprotection

## Abstract

Finding new therapies for Parkinson’s disease (PD) is a slow process. We assembled an international committee of experts to examine drugs potentially suitable for repurposing to modify PD progression. This committee evaluated multiple drugs currently used, or being developed, in other therapeutic areas, as well as considering several natural, non-pharmaceutical compounds. The committee prioritized which of these putative treatments were most suited to move immediately into pilot clinical trials. Aspects considered included known modes of action, safety, blood-brain-barrier penetration, preclinical data in animal models of PD and the possibility to monitor target engagement in the brain. Of the 26 potential interventions, 10 were considered worth moving forward into small, parallel ‘learning’ clinical trials in PD patients. These trials could be funded in a multitude of ways through support from industry, research grants and directed philanthropic donations. The committee-based approach to select the candidate compounds might help rapidly identify new potential PD treatment strategies for use in clinical trials.

## BACKGROUND

Multiple new therapies for Parkinson’s disease (PD) have emerged following the initial development of levodopa therapy, including dopamine receptor agonists, MAO-B inhibitors, COMT inhibitors and deep brain stimulation. These treatments predominantly still involve an approach of dopamine-replacement to reduce symptoms acutely, without achieving slowing of disease progression. Levodopa-based therapy is complicated by the emergence of motor fluctuations and dyskinesias, and has limited effects on a range of non-motor PD symptoms (e.g. cognition, postural stability) that are the source of significant morbidity. It may be possible to accelerate the process of identifying new PD treatments by repurposing medicines (drug repositioning) that are approved for other indications because of evidence that they may also have beneficial actions on PD progression.

In order to try and facilitate identification of such compounds for repositioning we have set up a Linked Clinical Trials initiative (LCT), a structured approach for accelerating new treatments for PD. This initiative arose for 4 reasons; 1) basic research breakthroughs regarding PD pathophysiology; 2) previous failure in developing treatments for PD progression; 3) the realisation that many agents in clinical use for other medical conditions may have off-target effects relevant to PD; and 4) the lengthy procedures needed to take a drug from the laboratory to the clinic.

Drug repositioning is highly attractive in that it partly focuses on drugs with regulatory-approved clinical safety data, and there are thousands of regulatory-approved biologically-active drugs already available. If these approved drugs include agents with efficacy in the treatment of PD progression, repositioning may represent a more efficient process [1–8] than original drug discovery [9]. Indeed, repositioning in some therapeutic areas has provided crucial strategic advances in the introduction of new treatments (e.g. see O’Connor and Roth [10]). The success rates of the drug repositioning approach and using drugs that have already passed Phase I safety/toxicology studies can approach 30% [11], and this represents a huge improvement on traditional forms of drug discovery, where typically the success rate is much less than 10% [12].

## WHAT DRUG REPOSITIONING HAS BEEN CONDUCTED IN PD TO DATE?

Clinical trials in PD using five different repositioned drugs have been undertaken, Exenatide [13], a GLP-1 agonist originally approved for use in Diabetes Type II, Pioglitazone [14], a glitazone originally approved for use in Diabetes Type II, Isradipine [15], a calcium channel blocker originally approved for use in hypertension, Deferiprone [16], an iron chelator originally approved for use in beta-thalassaemia and, Inosine [17], used in the SURE-PD study to raise serum and cerebrospinal fluid levels of urate (all identifiers for these clinical trials are listed in the references).

The recently-published Exenatide pilot clinical trial [18] apart from its clinical findings, also demonstrated the feasibility [19] of running a learning trial in a relatively small number of PD patients. Exenatide is an intervention originally targeted at Diabetes Type II but which was thought a priori to offer considerable potency and potential clinical benefit in the treatment of PD [20–25].

The notion that small screening studies for putative new treatments are applied to PD is not new. A series of futility studies were conducted some years ago with a similar aim; to determine cost-effectively whether a full Phase 3 clinical trial was merited for each agent tested. Notably, Coenzyme Q10, the neuroimmunophilin-ligand GPI-1485, creatine and minocycline were tested in 2 futility studies, that provided information suggesting that it was warranted to proceed with creatine [26–28]. Adding to this experience was the DATAOP study of vitamin E and selegiline initiated in the 1980’s [29]. Some of the data from DATATOP were used in the futility initiative planning, along with the CINAPS process that reviewed potential compounds [30]. This latter process published dossiers describing the attributes of 26 suggested putative new treatments for PD described as ‘potential neuroprotective agents to treat the symptoms and progression of Parkinson’s disease [30, 31].

We therefore sought to build on this initiative and felt that the best way to do this was to set up a formal international committee tasked to prioritize which putative treatments should move quickly into clinical trials.

## METHODS

We undertook an extensive review process of marketed drugs and drugs under development in many different therapeutic areas, as well as several natural non-pharmaceutical compounds. Essentially, this process involved rigorous and continual scrutiny of the results of an evolving wide range of pharmaceutical approaches used by basic and clinical researchers trying to influence cellular function and cellular protection in other key fields of medical research, most especially in ophthalmology, cardiology, metabolic diseases, and oncology. The aim was to identify agents that may modify the underlying pathogenesis in PD, and also to establish a process for technology transfer of new biochemical/pharmaceutical approaches where breakthroughs in other therapeutic areas could be rapidly applied to PD where there was a rationale to do so.

This process initially identified 72 potential new candidate therapeutic approaches for PD that addressed specific biochemical targets of interest. On closer scrutiny, these 72 candidates were reduced to 26, mainly on grounds of safety, blood-brain-barrier penetration, or commercial/patent issues. Succinct supporting dossiers (5–7 pages) were written describing the reasons why each of the 26 proposed interventions should be considered for entry into learning (pilot) clinical trials in PD patients.

These 26 dossiers were then assessed by an international committee of experts (see Appendix 1) with the aim being to prioritize the therapeutic candidates. In the days prior to this meeting, the committee members were first asked to pre-prioritize each of these 26 interventions in terms of their merit for entry into PD trials. The criteria employed during the prioritization included drug safety; passage of the drug across the blood-brain barrier; a mode of action suggesting that the drug might be effective in PD; the possibility to assess that the drug engages the target in the brain; and demonstrated effects in an animal model of PD. Regarding effects in animal models, drugs showing effect in more than one type (neurotoxin-, protein aggregate- or gene-based) of animal PD model were deemed more interesting. It was also viewed as an advantage if independent laboratories had published data on the same drug.

This initial triage process removed several of the proposed therapies in terms of insufficient support for their use in PD. In view of the multiple funding sources required to facilitate a considerable number of parallel learning trials in PD patients, representatives of key funding bodies were also invited to attend including NIH/NINDS, Van Andel Institute, The Michael J Fox Foundation for Parkinson’s Research, The Kinetics Foundation, The Parkinson’s Disease Foundation, Parkinson’s UK, and The Cure Parkinson’s Trust, as well as several patients with PD. A number of other major funding bodies (The Wellcome Trust, Medical Research Council, and the Technology Strategy Board), who were not able to attend the committee meeting were given in-person, detailed debriefings of the outcomes of the LCT committee meeting.

## RESULTS

The 26 candidate agents presented to the committee are shown in Table 1 together with their likely mode(s) of action and target pathway.

### Committee initial pre-prioritization of interventions

**Fig. 1:**
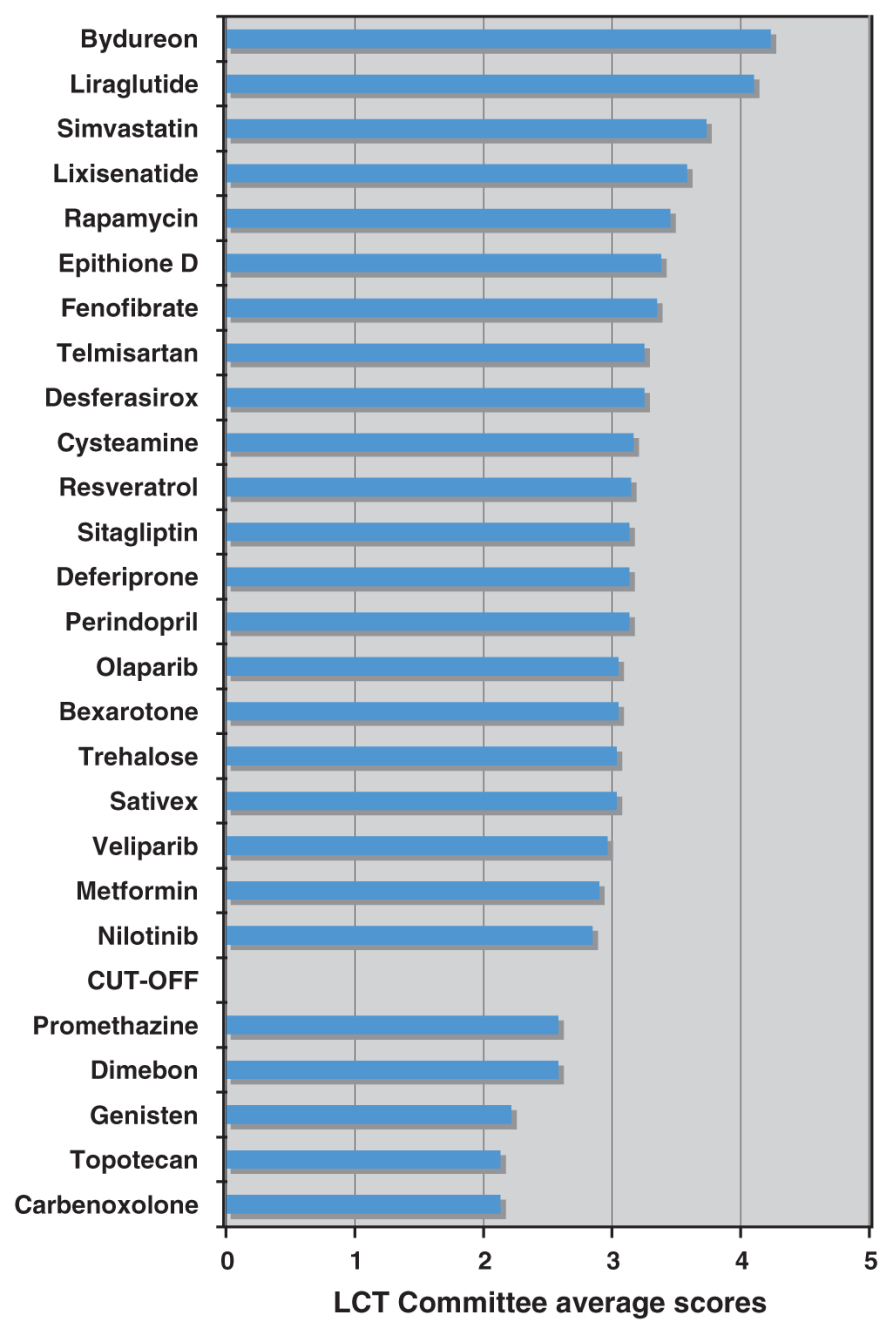
Pre-prioritization of the initial 26 candidate PD therapies for rapid translation to clinical trials.

The average pre-prioritization scores allocated by the LCT committee members (before their face-to-face committee meeting) are presented in Fig. 1. The X axis of Fig. 1 shows the average scores given by LCT committee members for each of the 26 proposed interventions assessed during the initial pre-prioritization phase (score ranges were 0 = lowest prioritization, to 5 = highest prioritization). Based on the average scores awarded, the committee reduced the number of interventions being considered at the subsequent face-to-face LCT committee meeting by only including compounds receiving strong pre-prioritization committee support. By this process, 5 of these interventions were triaged out on the basis of insufficient committee support for those proposed treatments.

The candidate treatments above the cut-off point on Fig. 1 were thus chosen for specific detailed discussion at the 2-day committee meeting where they were each assessed in terms of their various merits for testing in PD trials. These discussions led to a list of 7 prioritized interventions that were recommended for immediate entry into learning (pilot) PD clinical trials. The prioritization scores for these interventions (6 drugs & 1 natural compound) are listed in Table 2.

In addition, 5 other candidates (Rapamycin, Nilotinib, Cysteamine, Epithilone D and Resveratrol) were considered potentially interesting by the committee, but they were placed on a waiting list, subject to further information becoming available on them. The awaited information included impending results of a clinical trial using the same intervention in another therapeutic area, questions about physical characteristics of the interventions (such as blood-brain-barrier penetration) or further unpublished information (to be requested from their commercial owners) that might impact on the selection of the intervention, or subsequent trial design.

The 7 therapeutic candidates initially selected/prioritized are now being moved into learning (pilot) clinical trials involving a worldwide clinical trial network currently being established as part of the LCT initiative.

## DISCUSSION

With an increasing number of biochemical targets emerging as potentially relevant to PD progression comes a need to develop new approaches for identifying therapies that will engage those targets. Drug repositioning from existing therapeutics, or from interventions under development in other disease areas, represents an effective interim way forward whilst more specifically designed drugs can be identified, synthesized and brought through from pre-clinical studies. Since safety and toxicology data in humans is already known for these repositioned drugs, it means that success rates of such approaches can reach 30%, which is a vast improvement on traditional drug development methods [11, 12].

How can one best identify drugs suitable for repositioning for PD? We focused on current understanding of PD-relevant biochemical and pharmaceutical characteristics involving research that straddled recent advances in many other therapeutic areas. The LCT initiative aims to use methods of candidate identification as propounded by O’Connor and Roth [10].

As the success rates of Phase 2 clinical trials continue to fall [32], often through limitations of disease models, as well as questions to do with target validity [33], drug repositioning offers a promising way forward. We therefore anticipate improved success rates using results from high quality Phase 2 learning PD clinical trials, as envisaged here in the LCT initiative, to determine which candidate intervention should subsequently be progressed into Phase 3 trials.

Indeed, Sherer et al. [34] commented that ‘the PD research community needs to make a concerted effort to move beyond simply discovery targets and commit to providing compelling data for launching drug development initiatives around promising targets’. This is exactly what the LCT initiative aims to achieve. Newer paradigms involving open pharmaceutical innovation [35], which we have experienced at first hand, as well as NINDS and PPMI biomarker initiatives [34, 36] are likely to assist progress greatly.

In fact, since most drugs for use in neurology have been discovered empirically, it has been suggested that CNS diseases are far less suited to target-based approaches than other therapeutic areas [37]. The unpredictability of functional outcomes when dealing with highly interconnected metabolic networks can be usefully exploited for therapeutic discovery, with unanticipated relationships that emerge very frequently producing “off-target” actions that may lead to unexpected therapeutic actions of interventions [38, 39]. This is one reason why drug repositioning is growing in importance to supplement an arguably ailing innovation gap. Furthermore, for approved drugs the safety and toxicology have already been reviewed as acceptable by the regulators, meaning that subsequent development costs, timings, commercial risk and the chance of therapeutic failures are all reduced [1].

We prepared study dossiers for 26 potential interventions (Table 1). These agents included 3 GLP-1 agonists, as well as a gliptin (which slows the breakdown of natural GLP-1 amongst other actions), Metformin (an AMPK activator and GLP-1 activator), two iron chelators, a statin, a fibrate, an ACE inhibitor, an Angiotensin Receptor Blocker, mTOR inhibitors, PARP inhibitors, a Kinase inhibitor, a Microtubular stabilizer, a Retinoid, and a SIRT1 activator. During the preparation phase, combination therapies amongst these interventions, and in combination with other pharmaceuticals, was also considered but at this stage results from single agent therapies seems to be the more logical way forward.

Of the 26 interventions selected for committee evaluation, some were already known to influence multiple biochemical targets. Furthermore, some of these targets were known to be influenced by more than one of the drug candidates being considered. For example, in terms of drugs targeting G protein coupled receptors (GPCR), which include cyclic AMP receptors, there were 6 drugs considered by the committee (Rapamycin, 3 GLP-1 agonists, Sitagliptin, and Telmisartan).

Indeed in the past, NINDS undertook a similar process and created a series of excellent dossiers describing putative approaches for testing in PD (Table 3). Three of these CINAPS dossiers, describing Cystamine, Dimebon, and Sirolimus (Rapamycin), were related to interventions that were also amongst the 26 LCT dossiers, with one other (Candesartan) ‘replaced’ by Telmisartan as a preferred alternative within this drug class because of better pharmacokinetics (Telmisartan has the longest half life of any angiotensin receptor blocker, slow clearance from the brain, and some additional anti-inflammatory actions).

**Fig. 2:**
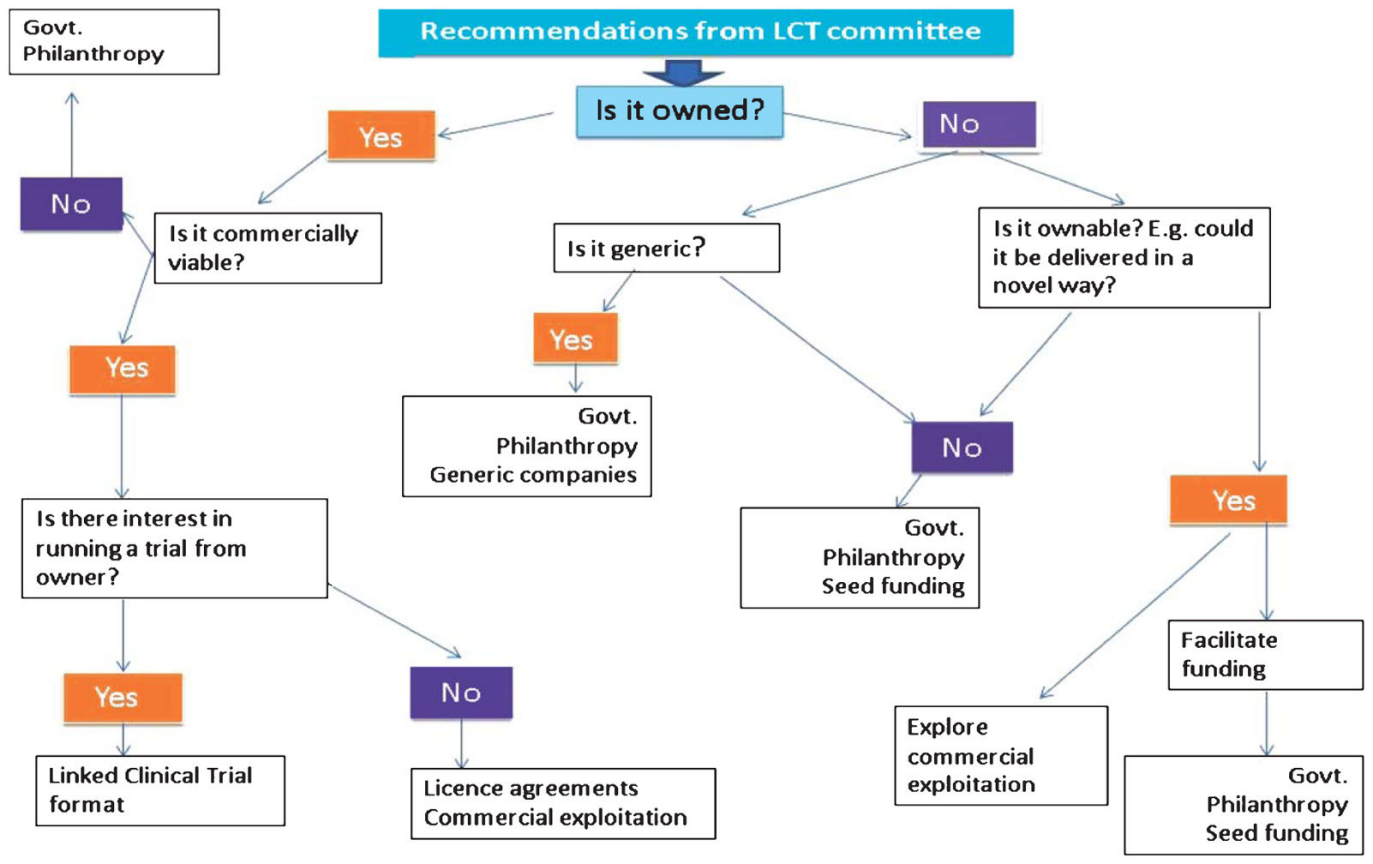
Funding strategies for treatments prioritized within the LCT initiative depending on ownership of individual interventions.

Some, but not all, of the candidates in the LCT initiative are owned by pharmaceutical companies which means that the future funding of any prioritized candidate might be influenced by ownership. So whether or not an agent is owned by a pharmaceutical agent, funding might well need to be secured from non-commercial sources (see Fig. 2) if the company does not wish to invest in any possible use of their agent in PD.

Also attending the LCT Committee meeting was an executive group comprising the main funding bodies that support basic and clinical research in PD, several of whom had already been funding pre-clinical laboratory studies involving candidates now being considered for pilot clinical studies. It is hoped that by involving them at this stage, that the funding of trials related to selected targets can happen quickly.

Across the treatments that were finally prioritized by the committee, it was recognised that here is an opportunity to harmonise the trial designs including control groups and outcome measures. An initiative like this also has the capacity to set up co-ordinated multi arm trials, as has been done in some cancer studies [40].

In conclusion, the intelligent use of drug repositioning is likely to represent a powerful bridging strategy for testing drugs active on multiple targets, some of which are directly relevant to the treatment of PD. The LCT initiative seeks to formalise this process and use a comprehensive approach that annually screens the literature for new interventions. The committee then prioritizes the agents for clinical trial evaluation through an on-going, practical head-to-head screening of important new approaches. By so doing, it is hoped that the identification of safe and effective new agents can be undertaken with greater speed, as we attempt to slow down or even halt the progression of this disorder.

## Figures and Tables

**Table 1:** Dossiers written on 26 candidate interventions for committee pre-prioritization

Intervention	Drug class/target pathway
Rapamycin	Rapalogue, mTOR inhibitor, Immunomodulator; FKBP12/mTORC1
Bydureon/Exenatide	GLP-1 agonist; target pathway, cAMP
Bydureon/Exenatide	GLP-1 agonist; target pathway, cAMP
Liraglutide	GLP-1 agonist; target pathway, cAMP
Liraglutide	GLP-1 agonist; target pathway, cAMP
Lixisenatide	GLP-1 agonist; target pathway, cAMP
Lixisenatide	GLP-1 agonist; target pathway, cAMP
Sitagliptin	Dipeptidyl (DPP-4) inhibitor, anti-inflammatory
Metformin	Biguanide, AMPK activator, mTOR inhibitor, stimulates GLP-1 release
Olaparib	Poly ADP ribose polymerase (PARP) inhibitor
Veliparib	Poly ADP ribose polymerase (both PARP1 and PARP2) inhibitor
Nilotinib	Selective c-ABl/Bcr-Abl kinase inhibitor
Deferasirox	Iron chelator; to target iron accumulation in substantia nigra, and oxidative stress also.
Deferiprone	Iron chelator; to target iron accumulation in substantia nigra, and oxidative stress also.
Cysteamine	Antioxidant, increases central BDNF, transglutaminase inhibitor
Epithilone D	Microtubular stabiliser
Trehalose	Natural non-reducing disaccharide, mTOR-independent activator of autophagy, antioxidant, promotor of protein disaggregation
Bexaratene	Retinoid
Simvastatin	Statin. Multiple biochemical actions unrelated to lipid lowering, including prevention of striatal dopamine depletion, restoration of striatal fibers and intracellular trafficking, reduction of aggregation of cellular alpha-synuclein, improvements in mitochondrial function through increased expression of PPAR-α and improvement of motor function
Fenofibrate	Fibrate, PPAR-α agonist (perhaps PPARγ and PPAR*δ* agonists also)
Perindopril	Angiotensin converting enzyme (ACE) inhibitor.
Telmisartan	Angiotensin receptor blocker (ARB); angiotensin II type I receptor
Sativex	Cannabinoid, anti-inflammatory; PPARγ/antioxidant properties
Carbenoxolone	Non-selective 11β-hydroxysteroid dehydrogenase inhibitor
Topotecan	Camptothecin; topoisomerase-1 and mitosis inhibitor; ubiquitin ligase
Genistein	Isoflavone phytoestrogen, Estrogen receptor beta agonist, antioxidant, PPARγ activator, tyrosine kinase inhibitor
Dimebon	Anti-histamine, weak NMDA antagonist, mitochondrial calcium homeostasis stabiliser, cholinesterase inhibitor, mTOR pathway inhibitor
Promethazine	Anti-histamine, NMDA receptor antagonist, mitochondrial membrane potential stabiliser
Resveratrol	Naturally occurring Polyphenol, Specific activator of SIRT1, Possible additional action on mTORC1, Possible direct action on PGC-1α, Antioxidant, Anti-inflammatory, Increases GLP-1 levels

**Table 2:** Outcome of final committee evaluation and prioritization

Intervention	Average Pre-prioritization scores (1 = lowest, 5 = highest)	Scores allocated at committee meeting (3 = lowest, 1 = highest)
Bydureon/Exenatide (2 patient groups selected)	4.16	1.5
Liraglutide (2 patient groups selected)	4.05	1.5
Lixisenatide (2 patient groups selected)	3.59	1.5
Deferiprone and Deferasirox	3.39	1.5
Simvastatin	3.69	1.5–2.0
Trehalose	3.24	1.5–2.1

**Table 3:** List of CINAPS ‘Compound Dossiers’ (January 2012), describing potential neuroprotective agents to treat the symptoms and progression of Parkinson’s disease

Acetyl-L-carnitine	Lipoic acid	Safinamide
Candesartan	Melatonin	Sirolimus
Celastrol	Memantine	Tamoxifen
Citicoline	MitoQ	Taurine
Clioquinol	Nisoxetine	Topiramate
Cystamine	Phenylbutyrate	Triacetyluridine
Geldanamycin	Pioglitazone	Valproic acid
Isradipine	Pramipexole	
Levetiracetam	Reboxetine	

## APPENDIX 1

### Founding members of the international linked clinical trials committee

Patrik Brundin
Roger A. Barker
P. Jeffrey Conn
Ted M. Dawson
Karl Kieburtz
Andrew J. Lees
Michael A. Schwarzschild
Caroline M. Tanner

### Executive committee/observers

Dr Wendy Galpern NINDS, NIH
Dr Beth-Ann Seiber NINDS, NIH
Dr Debra Babcock NINDS, NIH
Dr Maurizio Facheris Michael J Fox Foundation
Dr James Beck, Parkinson’s Disease Foundation
Dr Kieran Breen, Parkinson’s UK
Ken Kubota, Kinetics Foundation
Dr Brent Mulder, Van Andel Institute
Tom DeKoning, Van Andel Institute
Dr Martha Escobar, Van Andel Institute
Dr Susan Hoppough, Trinity Health, Grand Rapids, USA
Steve De Witt (Parkinson’s Advocate)
Mike McConnell (Parkinson’s Advocate)
Joy Duffen, Cure Parkinson’s Trust
Tom Isaacs, Cure Parkinson’s Trust
Helen Matthews, Cure Parkinson’s Trust
Dr Richard K.H. Wyse, the Cure Parkinson’s Trust

Unable to attend as observers were a number of other major funding bodies (e.g. The Wellcome Trust, Medical Research Council, and the Technology Strategy Board), who were subsequently given in-person, detailed accounts of the outcomes of the LCT committee meeting.
